# Epithelioid angiomyolipoma mimicking adrenal cortical carcinoma: A diagnostic pitfall

**DOI:** 10.3892/ol.2015.3543

**Published:** 2015-07-29

**Authors:** HANNA KOMAROWSKA, KATARZYNA BEDNAREK-RAJEWSKA, MARCIN KAŃSKI, MAŁGORZATA JANICKA-JEDYŃSKA, PAWEŁ GUT, MAREK RUCHAŁA

**Affiliations:** 1 Department of Endocrinology, Metabolism and Internal Medicine, Poznań University of Medical Sciences, Poznań 60-355, Poland; 2 Department of Clinical Pathomorphology, Poznań University of Medical Sciences, Poznań 60-355, Poland; 3 Department of Geriatric Medicine and Gerontology, Poznań University of Medical Sciences, Poznań 60-355, Poland

**Keywords:** epithelioid angiomyolipoma, adrenal cortical carcinoma, histopathological diagnosis

## Abstract

Epithelioid angiomyolipoma (EAML) is a rare mesenchymal neoplasmic variant of angiomyolipoma characterized by aggressive growth and unpredictable outcome. Cases of local recurrence and distant metastasis have been described. The histopathological diagnosis may be difficult, as EAML often mimics other neoplasms. This is the case report of a 39-year-old male patient with EAML, which was initially diagnosed as adrenal cortical carcinoma, due to the lack of cooperation between clinicians and pathologists.

## Introduction

Angiomyolipoma (AML) is a benign mesenchymal neoplasm composed of smooth muscle cells, adipose tissue and thick-walled dystrophic blood vessels arising from perivascular epithelioid cells. Epithelioid AML (EAML) is a variant of AML, which is characterized by more aggressive growth and a less favorable clinical outcome. EAML is composed of cells arranged in nests, sheets or broad alveoli surrounded by vascular septae. The cells within the tumor may be spindled or epithelioid, with clear or eosinophilic cytoplasm. There are also tumors that present with numerous pleomorphic multinucleated cells. Cases of local recurrence and metastasis, mainly in the liver, lymph nodes, lungs, other retroperitoneal organs and bones, have been previously described. The histopathological diagnosis of this tumor may be difficult, as EAML often mimics other neoplasms (1–5). This is the case report of a 39-year-old male patient with EAML, which was initially diagnosed as adrenal cortical carcinoma (ACC), due to the lack of proper cooperation between clinicians and pathologists.

## Case report

### 

#### Patient

In December 2013, a 39-year-old male patient diagnosed with cancer of the right adrenal gland was admitted to the Department of Endocrinology, Metabolism and Internal Medicine, Poznań University of Medical Sciences (Poznań, Poland) for further treatment.

#### Medical interview

The patient presented 3 years prior with a tumor in the right perirenal area, which was resected and diagnosed as a neoplasm of uncertain origin, possibly AML or metastatic melanoma. Following surgery, the patient underwent 6 months of oncological follow-up. In September, 2013 the patient was admitted to the hospital with low-grade fever and abdominal pain. A computed tomography (CT) scan of the abdomen revealed a large retroperitoneal tumor in close proximity to the right kidney. The patient underwent surgery in October, 2013 and the tumor along with the right kidney were resected.

#### Macroscopic findings

A tissue specimen described as an adrenal gland tumor measuring 16.0×9.0×10.0 cm was sent to the Department of Pathology (Poznań University of Medical Sciences). On sectioning, the tumor was polycystic, tan-brown, including a prominent necrotic and hemorrhagic area measuring 10.0×8.0×6.0 cm. A fragment of orange-colored tissue was identified, measuring 1.1×1.5×0.7 cm, which was considered to be residual adrenal gland tissue. The tumor infiltrated through the capsule of the kidney, whereas the remainder of the kidney, renal pelvis, ureter and blood vessels presented no significant macroscopic findings.

#### Microscopic findings

**Figure 1.**
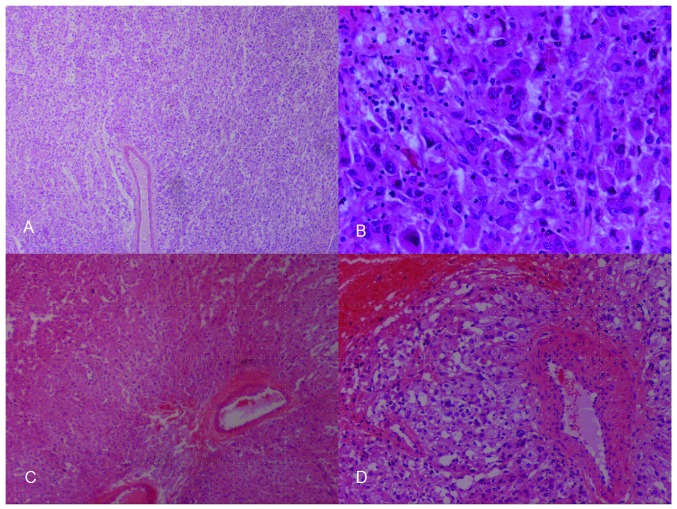
Histopathology. (A) EAML is composed of nests and sheets of cells separated by thin vascular septae (HE; magnification, ×100). (B) Sheets of spindled and plump epithelioid cells with eosinophilic cytoplasm (HE; magnification, ×200). (C) EAML with the presence of focal dysmorphic vessels within the tumor (HE; magnification, ×100). (D) EAML exhibiting sheets of pleomorphic epithelioid cells and thick-walled dysmorphic vessels (HE; magnification, ×200). EAML, epithelioid angiomyolipoma; HE, hematoxylin and eosin.

**Figure 2.**
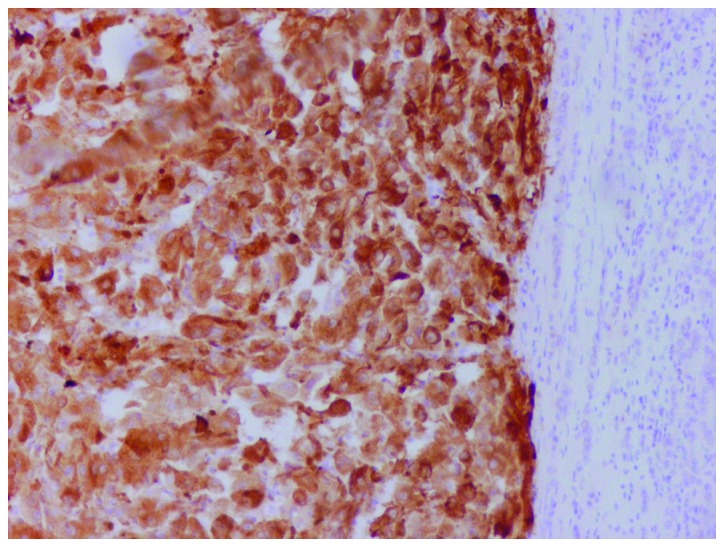
Immunohistochemistry. The tumor exhibited diffuse cytoplasmic reactivity for human melanoma black-45 (magnification, ×100).

**Figure 3.**
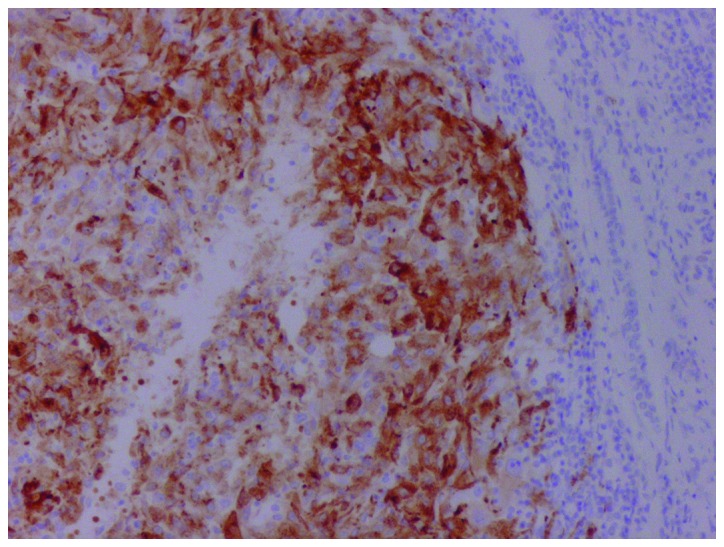
Immunohistochemistry. The tumor exhibited diffuse cytoplasmic reactivity for Melan-A (magnification, ×100).

**Figure 4.**
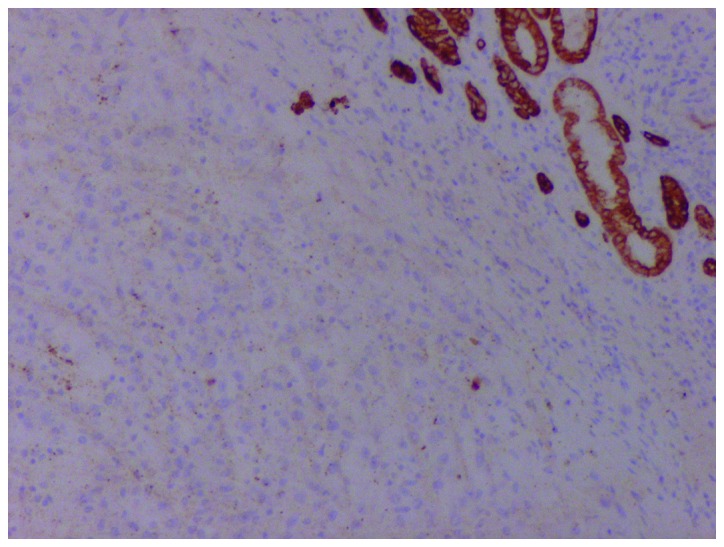
On immunohistochemical examination, the tumor tissue was negative for keratins (magnification, ×100).

The histopathology report described a tumor composed mostly of epithelioid cells with high nuclear pleomorphism, which formed solid areas and smaller nests surrounded by numerous blood vessels. The tumor infiltrated the capsule and cortex of the kidney and the surgical margins were positive for tumor cells. Necrotic and hemorrhagic areas were identified within the tumor, whereas the mitotic index was 5–7 mitoses per 10 high-power fields (Fig. 1). The tumor exhibited a strong immunoreactivity for vimentin, human melanoma black-45 (Fig. 2) and Melan-A (Fig. 3), whereas it was negative for keratins (Fig. 4), S100, synaptophysin and chromogranin A. The histopathology report concluded that, despite the mildly atypical immunohistochemical examination results, the microscopic characteristics strongly suggested the diagnosis of ACC. This diagnosis was also supported by the fact that the tumor was sent to the Department of Pathology as an adrenal tumor and the gross examination also strongly suggested that it originated from the adrenal gland.

The patient was diagnosed with adrenal gland carcinoma and was scheduled to receive mitotane treatment. Prior to the onset of treatment, the detailed medical history of the patient was recorded and all the necessary tests and examinations were performed. The patient had no known family history of endocrine malignancies.

#### Physical examination

Upon admission, the general condition of the patient was good, with the exception of complaints regarding minor pain in the muscles of the upper extremities. On physical examination there were no significant findings, such as signs of hypercortisolemia. The laboratory tests revealed dyslipidemia, marginally elevated creatinine levels and iron deficiency. The radiological imaging findings were unremarkable, revealing intact bilateral adrenal glands and absence of the right kidney. On bone scintigraphy analysis, there was increased tracer concentration in the left humeral joint (there was a query regarding previous arm luxation) and focal changes in the ribs suggestive of either osteoporotic fractures or metastases.

Due to the unexpectedly good condition of the patient, reassessment of the initial adrenal cancer diagnosis was considered, starting with the re-evaluation of the pathological findings. The pathologists were informed that the patient underwent surgery for a kidney tumor in 2010 in another hospital, and that the specimen sent to the Department of Pathology was a kidney tumor rather than an adrenal gland tumor. The pathologists also retrieved the previous histopathology report. Taking into consideration all the available information, the pathologists decided to perform additional immunohistochemical analyses, ultimately changing the previous diagnosis of adrenal gland carcinoma to EAML. Considering that this was a recurrent tumor with a diameter of >10 cm, composed mostly of atypical epithelioid cells with increased mitotic activity and infiltration of the perinephric fat, the pathologists were unable to predict the benign or malignant behavior of the tumor, as such lesions may recur and even metastasize. The patient was discharged in a good general condition. Further oncological follow-up was recommended.

Written informed consent for the publication of this case report was obtained from the patient.

## Discussion

The epithelioid variant of AML was first described in 1995. According to the available data, only ~160 cases of EAML had been described up to 2014. There are several characteristics distinguishing EAML from classic AML. The majority of EAMLs are larger compared with AMLs (the average size of the tumor at diagnosis has been reported to be 8.6 cm) (3), are mainly composed of epithelioid cells, and often lack the typical fat tissue component characteristic for this type of neoplasm. The malignant behavior, recurrence and metastasis rates of EAMLs cannot be easily determined. The average age at diagnosis of AML is 40 years (6,7).

EAML may be difficult to diagnose, as it may mimic other neoplasms on radiological imaging and histopathological examination. EAML is known to display a variable immunohistochemical phenotype and, therefore, may be misdiagnosed as renal cell carcinoma, ACC, hepatocellular carcinoma, epithelioid variant of malignant melanoma or hepatoblastoma. A comparison of the immunohistochemical profile of EAML, the present case and ACC is presented in Table I (3,8,9).

The coexistence of EAML with tuberous sclerosis is the most essential characteristic of its clinical presentation. Tuberous sclerosis is a genetic disorder characterized by mental impairment, autism, seizures and the presence of various tumors. In certain cases, the disease is otherwise asymptomatic, apart from the mass effect of the tumor as it grows to a larger size (10).

According to Nese *et al* (7), the coexistence of EAML with tuberous sclerosis or AML, the presence of tumor necrosis, infiltration of the tumor beyond the renal parenchyma, infiltration of the renal vein and ‘carcinoma-like’ growth pattern are characteristics associated with an unfavorable prognosis. The main treatment is the resection of the tumor. Alternative treatment strategies include chemoradiation or arterial embolization of the tumor. There have also been attempts to use mammalian target of rapamycin (mTOR) inhibitors, including temsirolimus and everolimus (11).

ACC is a rare epithelial tumor, which is derived from the cortex of the adrenal gland, with a frequency of 1–12 cases per million (12–14). ACC most commonly occurs in patients aged 40–60 years and in children aged <5 years (12,14) and its mean diameter ranges between 5 and 20 cm (14). ACC is usually a sporadic tumor; however, it may occasionally be associated with the Li-Fraumeni syndrome, multiple neuroendocrine neoplasia type 1, or the Carney complex (14). The clinical manifestations of this neoplasm depend on its hormonal activity, which may be present in 60–80% of adult patients displaying symptoms of Cushing's syndrome or androgenization (14). In rare cases of ACC without hormonal activity, the symptoms are associated with the presence of the tumor mass or the presence of distant metastases and disseminated neoplastic disease (14). There are four clinical stages of ACC (15).

The histopathological diagnosis of ACC may be difficult. There is currently no consensus regarding decision-making as to whether ACCs are benign or malignant. The diagnosis is most commonly based on the Weiss criteria with Aubert's modifications (Table II) (16).

The treatment of ACC includes surgery and mitotane therapy, which is commonly associated with side effects, requiring a reduction of the dose. In particular cases, it may be necessary to add chemotherapy with etoposide, doxorubicin and cisplatin or streptokinase. The prognosis for patients with ACC is unfavorable (17).

The diagnosis of EAML and ACC may be challenging. Cases of stage-1 and −2 ACC without hormonal activity may be clinically identical to EAML. In the present case, the age of the patient was not a valuable indicator, as it was typical for both types of neoplasm.

It was significant for the endocrinologist that the general health condition of the patient was surprisingly good, despite the fact that the diagnosis was ACC relapse. There were also doubts regarding the results of the histopathological examination after the first operation. Additionally, the presence of both adrenal glands on CT imaging caused the clinician to consider verifying the second histopathological diagnosis.

The present case stresses the significance of the close cooperation between clinicians and pathologists. It is necessary to provide pathologists with all the available medical history and physical examination results. In the present case, the pathologist was wrongly informed of the tumor site (the tumor was previously described and sent to the Department of Pathology as an adrenal neoplasm). There was also no information regarding the previous histology results, which considered the diagnosis of EAML or metastatic melanoma. This information would not only help the pathologist reach an accurate diagnosis, but would also save time and money spent on immunohistochemical assays. The thorough review of the medical history of the patient by the clinician helped avoid further unnecessary toxic and costly treatment.

## Figures and Tables

**Table I.** Immunohistochemical comparison of EAML, the present case and ACC.

Immunohistochemical markers	EAML	Present case	ACC
S-100	−/+	–	+/−
Melan-A	+	+	+
HMB-45	+	+	–
CD117	+	–	−/+
CD63	+	Not performed	NR
Keratins	−/+	–	−/+
SMA	+/-	+^a^	–
Desmin	−/+	–^a^	–
Vimentin		+	+
Synaptophysin		–	+
Calretinin			+
D2-40			+
Inhibin			+
SF1			+
Pankeratin			+/-

a Immunohistochemical staining for SMA and desmin was conducted to verify the results of the first histopathological examination. +, positive; -, negative; +/-, may show focal positivity; -/+, sometimes totally negative; NR, not reported; HMB-45, human melanoma black-45; SMA, smooth muscle actin; SF1, steroidogenic factor 1; EAML, epithelioid angiomyolipoma; ACC, adrenal cortical carcinoma.

**Table II.** Weiss criteria with Aubert's modifications. The threshold for identifying malignant behaviour is ≥3.

No.	Criteria
1	Nuclear grade by Fuhrman (III/IV)
2	Mitotic index (>5/50 high-power fields)
3	Atypical mitoses
4	Clear cells (<25%)
5	Diffuse architecture (>33%)
6	Necrosis
7	Venus invasion
8	Sinusoidal invasion
9	Capsular invasion

## Abbreviations

AML: angiomyolipoma
EAML: epithelioid angiomyolipoma
ACC: adrenal cortical carcinoma
